# Inflammatory Forms of Cardiomyocyte Cell Death in the Rat Model of Isoprenaline-Induced Takotsubo Syndrome

**DOI:** 10.3390/biomedicines11072060

**Published:** 2023-07-21

**Authors:** Sonia Borodzicz-Jażdżyk, Agnieszka Kołodzińska, Katarzyna Czarzasta, Małgorzata Wojciechowska, Renata Główczyńska, Benedykt Szczepankiewicz, Liana Puchalska, Grzegorz Opolski, Agnieszka Cudnoch-Jędrzejewska

**Affiliations:** 1 Chair and Department of Experimental and Clinical Physiology, Laboratory of Centre for Preclinical Research, Medical University of Warsaw, 1b Banacha Street, 02-097 Warsaw, Poland; 2 1st Chair and Department of Cardiology, Medical University of Warsaw, 1a Banacha Street, 02-097 Warsaw, Poland; 3 Department of Pathology, Medical University of Warsaw, 7 Pawińskiego Street, 02-106 Warsaw, Poland

**Keywords:** Takotsubo syndrome, necroptosis, pyroptosis, inflammation, ovariectomy

## Abstract

Takotsubo syndrome (TTS) is associated with inflammatory response, therefore the aim of the study was to evaluate the presence and dynamics of inflammatory-associated forms of cell death, necroptosis, and pyroptosis in the female rat model of isoprenaline (ISO)-induced TTS. TTS was induced in female Sprague Dawley rats (n = 36) by ISO 150 mg/kg intraperitoneally. Animals were divided into four groups: TTSO (TTS+ovariectomy; n = 10), TTSP (TTS+sham operation; n = 10), CO (0.9% NaCl+ovariectomy; n = 8), CP (0.9% NaCl+sham operation; n = 8). Histopathological analysis, evaluation of plasma concentration, and myocardial expression of pyroptosis- and necroptosis-associated proteins were performed. TTSO and TTSP groups had higher plasma concentrations of interleukin-1β in comparison with the controls. Low myocardial protein expression of mixed lineage kinase domain-like pseudokinase (MLKL), caspase-1 (Casp-1), and calcium/calmodulin-dependent kinase type II isoform delta (CAMKIIδ) was visible 6 and/or 12 h post-ISO. Twenty-four hours post-ISO, high myocardial and vascular protein expression of CAMKIIδ was visible in TTSO but not TTSP rats, while high myocardial expression of MLKL and Casp-1 was visible both in TTSO and TTSP rats. The course of TTS is associated with activation of inflammatory-associated programmed cell death, necroptosis, and pyroptosis, therefore inflammation may be a primary response occurring simultaneously with cardiomyocyte death in TTS.

## 1. Introduction

In cardiovascular diseases, several forms of myocardial cell death are present, including apoptosis, necroptosis, mitochondrial-mediated necrosis, pyroptosis, ferroptosis, and autophagic cell death [1]. The death of cardiomyocytes disturbs cardiac contractility and promotes adverse cardiac remodeling, which, as a consequence, serves as a pathophysiological background for the development of cardiac dysfunction and heart failure (HF) [1,2]. Apoptosis and autophagy are processes that do not cause the rupture of the cell membrane of the cardiomyocytes. On the other hand, necroptosis and pyroptosis are associated with the disruption of the cell membrane, which leads to the release of cytoplasmic contents to the bloodstream, including inflammatory cytokines. The release of inflammatory cytokines from the disrupted cells serves as a stress signal, triggering a cascade of inflammatory response and protective mechanisms [1]. Necroptosis is a regulated form of cell death, mediated by death receptors, including tumor necrosis factor receptor 1 (TNFR1) and tumor necrosis factor-related apoptosis-inducing ligand (TRAIL), and Fas receptors [3,4]. Binding a ligand to TNFR1 results in the activation of the necroptotic pathway through RIP1 (receptor-interacting protein kinase 1), RIP3 (receptor-interacting protein kinase 3), and MLKL (mixed lineage kinase domain-like). RIP1 and RIP3 are known to form a RIP1/RIP3 complex, which is called a necrosome. Therefore, MLKL oligomerizes, translocates to the plasma membrane, and induces ion channel dysfunction and ion imbalance, which finally induces necroptosis [3,4]. It has been proven that the kinase activity of RIP3, the oligomerization of MLKL, and the formation of necrosome are essential in the necroptosis signaling pathway [3,4]. Pyroptosis is another form of programmed cell death associated with an inflammatory response, which can be induced by oxidative stress, inflammation, and hyperglycemia and is essential for controlling microbial infections [5]. In caspase-1 dependent pyroptosis, the pathogen-associated molecular patterns (PAMPs) and danger-associated molecular patterns (DAMPs) activate the inflammasome, including NLRP3, AIM2, or pyrin. NLRP3 oligomerizes and activates caspase-1 (Casp-1) and therefore initiates the maturation of proinflammatory cytokines such as IL-1β and IL-18, which are therefore released due to cell membrane disruption.

Takotsubo syndrome (TTS) is an acute HF syndrome that occurs mainly in postmenopausal women and is usually triggered by emotional or physical stress [6,7,8,9]. The pathogenesis of TTS has not yet been fully explained, although autonomic nervous system disbalance appears to play a crucial role [6,8]. Since TTS occurs mainly in postmenopausal women, the lack of female sex hormones is also thought to play an important role in the pathogenesis of TTS [10,11]. The established experimental model of TTS is induced by the administration of catecholamines, predominantly isoprenaline (ISO), and the development of the disease is diagnosed with the presence of typical LV wall motion abnormalities [12,13,14,15,16].

TTS is characterized by acute myocardial inflammatory cell infiltration, myocardial edema, and an increase in systemic proinflammatory cytokines, which may even persist for more than 5 months from the onset of the disease [17,18]. Although TTS has previously been considered a transient form of acute HF syndrome, long-term observation of these patients has revealed persisting HF symptoms with concomitant functional impairment, including reduced peak oxygen consumption, impaired global longitudinal and apical circumferential strain, increased native T1 mapping, and impaired cardiac energetic status [19]. Moreover, the residual high inflammatory response (defined as C-reactive protein > 19 mg/L at discharge) in patients with TTS is associated with a lower LV ejection fraction at follow-up and increased cardiac late mortality; it is also an independent predictor of cardiac death or hospitalization for HF [20].

Despite the fact that TTS occurs mostly in postmenopausal women, the vast majority of experimental studies on TTS are performed on male rats [13,14,21,22]. Under experimental conditions, menopause in rodents is simulated by a bilateral ovariectomy. We have therefore developed the first experimental model of TTS induced by ISO specifically in ovariectomized rats [23].

To date, only the presence of apoptosis has been proven in the course of experimental and clinical TTS [13,24,25]. In the context of the latest reports emphasizing the key role of inflammation in the acute phase and outcomes of TTS, the assessment of the presence of inflammatory-related cardiomyocyte cell death appears to be crucial for understanding the pathophysiology of the disease with a potential outlook for targeted pharmacotherapy. In our previous work, in a female rat model of TTS, we observed caspase-3 activity as an indicator of apoptosis in cardiomyocytes 24 h after ISO administration. Similarly, we observed apoptosis in mononuclear cells at 24–72 h and 7 days after ISO administration and in endothelial cells of vessels at 7 days post-ISO. Trichrome blue staining also showed mild fibrosis, which is characteristic of TTS, during both the acute and recovery phases. As a result, our research aimed to explore the presence of other forms of cell death throughout the course of TTS [13,26]. The aim of the current study was to evaluate the presence and dynamics of inflammatory-related types of cell death, namely necroptosis and pyroptosis, in the experimental menopause female rat model of isoprenaline (ISO)-induced Takotsubo syndrome.

## 2. Methods

The study was performed on 9-week-old female Sprague Dawley rats (SPRD/Clzd; n = 36) weighing 178.8 ± 12.4 g. The rats were housed in stable controlled conditions (temperature 22–25 °C; humidity 40–60%; 12 h light–dark cycle), with unlimited access to food and water. All experimental procedures were approved by the Local Animal Research Ethics Committee (No. 162/2016) and were consistent with Directive 2010/63/EU of the European Parliament and of the Council of 22 September 2010 on the protection of animals used for scientific purposes.

### 2.1. Ovariectomy and Sham Operation

Eighteen rats were subjected to bilateral ovariectomy at 9 weeks of age. The procedure was performed under general anesthesia (ketamine 75 mg/kg b.wt i.p. + xylazine 7 mg/kg b.wt i.p.). The rats were positioned on their back and a midline cut was made in the hypogastric region. The fallopian tubes were sutured in the distal part and the ovaries were cut. The wound was closed with a surgical suture (Vicryl 6.0, Ethicon, Somerville, MA, USA). Another eighteen rats at 9 weeks of age underwent a sham operation under general anesthesia (ketamine 75 mg/kg b.wt i.p. + xylazine 7 mg/kg b.wt i.p.) with a similar technique, however without the removal of the ovaries.

After each surgical procedure, all of the animals received postoperative care for the subsequent 2 days in the form of analgesic treatment (paracetamol 300 mg/kg b.wt administered in drinking water) and antibiotic therapy (enrofloxacin 5 mg/kg b.wt subcutaneously).

### 2.2. Induction of Takotsubo Syndrome

In twenty rats, three weeks after the ovariectomy or sham surgery, a single ISO dose of 150 mg/kg b.wt was injected i.p. to induce the experimental model of TTS, as described elsewhere [13]. After the injection of ISO, the rats were housed in individual cages to facilitate the observation of the condition of the animals under the influence of ISO. Sixteen control rats received an i.p. injection of 1 mL of saline.

### 2.3. Echocardiographic Examinations, Blood Collection, and Tissue Harvesting

To diagnose TTS, transthoracic two-dimensional echocardiography was performed using the Vivid i/Vivid q device (GE Medical Systems, Milwaukee, USA) with a pediatric cardiac sector array transducer (frequency range: 4.5–11.5 MHz, field of view 90°, depth of field 12 cm) in accordance to methodology used in other studies on experimental models of TTS [13,14]. Immediately prior to the examination, the rats were anesthetized by an injection of ketamine 75 mg/kg b.wt i.p. + xylazine 7 mg/kg b.wt i.p. The animals were laid in the left decubitus position on a bench. TTS was diagnosed by the presence of characteristic regional LV wall motion abnormalities; specifically: apical akinesia (aTTS), basal akinesia (bTTS), mid-basal akinesia (mbTTS), and both apical and basal akinesia (mixTTS) [14]. To characterize the dynamics of cell death-related protein expression over time, the echocardiographic examination, blood collection, and heart collection were performed after 6, 12, 24, and 72 h post-ISO injection. In control rats, the echocardiographic examination, blood collection, and heart collection were performed after 24 h post-saline administration.

### 2.4. Study Groups

The study groups were as follows:

TTSO—ovariectomized Sprague Dawley female rats with ISO-induced TTS (n = 10);
TTSP—sham-operated Sprague Dawley female rats with ISO-induced TTS (n = 10);
CO—control ovariectomized Sprague Dawley female rats injected with 0.9% NaCl (n = 8);
CP—control sham-operated Sprague Dawley female rats injected with 0.9% NaCl (n = 8).

### 2.5. Blood Collection and Heart Harvesting

At the end of each echocardiographic examination and under general anesthesia (ketamine 75 mg/kg b.wt i.p. + xylazine 7 mg/kg b.wt i.p.), 4 mL of blood from the RV was collected through a needle inserted in the third intercostal space into tubes with EDTA-K2 as an anticoagulant. The blood was then centrifuged at 1600× *g* for 15 minutes at 4 °C and the plasma was pipetted for biochemical tests. Immediately after collecting the blood, the animals were euthanized by i.p. administration of 267 mg/kg b.wt pentobarbital sodium + 53.4 mg/kg b.wt pentobarbital and, immediately after administration of anesthetics (without preceding perfusion of the rat with formaldehyde solution), the hearts were taken for further histopathological assessment. The hearts were placed in a sterile 50 mL container filled with 4% formaldehyde solution, which was filled to 3/4 of its capacity. This allowed for complete saturation of the cardiac muscle with the formaldehyde solution.

### 2.6. Histopathological Analysis

The hearts were cut into basal, mid-ventricular, and apical segments. The segments obtained were embedded in paraffin and cut into 4 μm sections and hematoxylin and eosin staining was performed. The apex, mid-ventricular, and basal segments of the LV and RV were analyzed, with an assessment of the endocardial, muscular, and epicardial layers of the LV wall. At 6, 12, and 24 h post-ISO injection, sections were evaluated at a magnification of 200×, while at 72 h post-ISO administration, the sections were assessed at a magnification of 40×.

Histopathological analysis included the assessment of interstitial edema, intracellular cardiomyocyte edema, endocardial and perivascular edema, the presence of blurred contours, the vacuolization of the cardiomyocytes, the presence of diffuse infiltration of inflammatory cells, the presence of hemorrhages and endocardial protrusions, and injury of the cardiomyocytes. The term foci of cardiomyocyte injury (FCI) was used to define focal cardiomyocyte injury accompanied by infiltration of inflammatory and fibroblast-like cells. Quantitatively, the number of FCIs was assessed per layer separately in the apical, mid-ventricular, and basal segments of the LV and RV. Arteritis was defined as disruption or destruction of the vessel wall with endothelial damage, extensive inflammatory infiltrations of media and adventitia, fibrin deposits, and red blood cell extravasation.

### 2.7. ELISA Analysis

The plasma levels of N-terminal pro B-type natriuretic peptide (NT-proBNP), troponin I (TnI), interleukin 1 beta (IL-1β), interleukin 18 (IL-18), casp-1, MLKL, RIP3, and calcium/calmodulin-dependent kinase type II isoform delta (CAMKIIδ) were evaluated using ELISA immunoassay kits (Sunred Biological Technology Co., Ltd., Shanghai, China, no. SRB-T-81148, SRB-T-80964, 201-11-0120/SRB-T-83324, SRB-T-82112, 201-11-2701, SRB-T-85954; FineTest, P. R. China no. ER0446; Invitrogen, Thermo Fisher Scientific, Waltham, MA, USA no. KRC2341).

### 2.8. Immunohistochemistry

Paraffin-fixed heart portions were sectioned into 3 µm thick slices, then deparaffinized and blocked with 3% hydrogen peroxide (peroxidase-blocking reagent, Dako, Santa Clara, CA, USA). To expose the target proteins, sections were heat treated in a Tris/EDTA buffer (target retrieval solution, Dako, Santa Clara, CA, USA). Then, staining was performed with the following antibodies: rabbit anti-caspase 1 antibody (ab74279; ABCAM, Cambridge, UK); rabbit anti-CaMKIIδ antibody (ab191588; ABCAM, UK); rabbit anti-MLKL (ab196436; ABCAM, UK); rabbit anti-RIPK3 (ab56164; ABCAM, UK). Rabbit LINKER reagents (Dako) were used to enhance the signal of the rabbit primary antibody. Signal detection was accomplished with goat anti-rabbit horseradish peroxidase (HRP)-conjugated secondary antibodies (Dako). 3,3′-diaminobenzidine (Dako) was used as the chromogen. Finally, the tissues were contrasted with hematoxylin. Negative controls were prepared by omitting the primary antibodies. Immunohistochemically stained slides were scanned with a Hamamatsu NanoZoomer 2.0-HT scanner, viewed using the NDP.view2 software, and then subjectively assessed. The immunohistochemical reactions were classified on a four degree scale: 0, complete lack of reaction; 1, negative reaction equal to the level of the background; 2, positive reaction stronger than the background (low expression); 3, positive strong immunohistochemistry reaction (high expression).

### 2.9. Statistical Analysis

Statistical analysis was performed using Statistica 13.3 software. In each time interval, the examined series TTS was diagnosed in n < 5 individuals; therefore, the statistical analysis of echocardiographic and biochemical parameters was performed for the total TTSO and TTSP groups. Comparisons of mean values of each parameter were performed using independent sample *t*-test or one-way ANOVA for normal distributions and non-parametric tests for distributions other than normal (ANOVA Kruskal–Wallis and Mann–Whitney U tests). The assessment of distribution was made using the Shapiro–Wilk W test and the homogeneity of variance was assessed using Levene’s test (ANOVA). All values were presented as mean ± SD. The differences were considered significant if *p* < 0.05.

## 3. Results

### 3.1. Echocardiographic Analysis

Echocardiographic analysis revealed the presence of LV segmental wall motion abnormalities typical for TTS in the TTSO and TTSP groups as early as 6 h post-ISO. In the TTSO group, the following variants of LV wall motion abnormalities were observed: aTTS (n = 2), bTTS (n = 4), mbTTS (n = 2), and mixTTS (n = 2). In the TTSP series, we also observed all types of TTS, including aTTS (n = 4), bTTS (n = 2), mbTTS (n = 3), and mixTTS (n = 1). The measurements of the fractional area change (FAC), fractional shortening (FS), LV end-diastolic area (LVEDA), LV end-diastolic dimension (LVEDD), and LV end-systolic area (LVESA) in the TTS and control rats are presented in Table 1. The rats from the CO and CP groups did not show any LV wall motion abnormalities. Echocardiographic parameters of cardiac function in TTSO and TTSP groups in different time points after ISO administration are presented in Supplementary Table S1.

### 3.2. Histopathological Analysis of Female Rat Hearts

The histopathological findings described below affected the endocardium and myocardium of all the segments of the heart, including the basal, the mid-ventricular, and the apical segments. Histopathological findings from the TTSO, TTSP, CO, and CP groups are summarized in Table 2.

**Figure 1:**
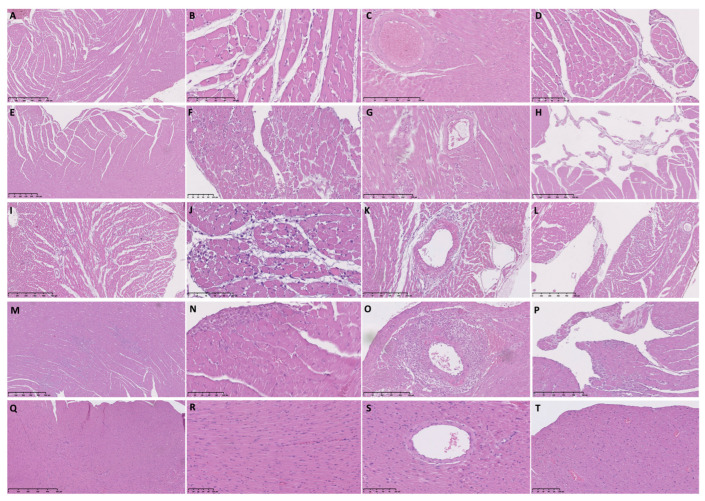
Histopathological findings in ovariectomized female Sprague Dawley rats with Takotsubo syndrome and ovariectomized control rats. (**A**) TTSO, 6 h post-ISO, apical LV segment. Slight interstitial edema is visible; (**B**) TTSO, 6 h post-ISO, apical LV segment. Interstitial edema, cardiomyocyte injury, and diffuse rare infiltration of inflammatory cells are present; (**C**) TTSO, 6 h post-ISO, mid-ventricular LV segment. Slight perivascular edema is visible; (**D**) TTSO, 6 h post-ISO, apical LV segment. Endocardial protrusion; (**E**) TTSO, 12 h post-ISO, mid-ventricular LV segment. Interstitial edema and slight endocardial edema are visible; (**F**) TTSO, 12 h post-ISO, mid-ventricular LV segment. Interstitial edema, diffuse inflammatory infiltration, and vacuolization of cardiomyocytes are present; (**G**) TTSO, 12 h post-ISO, apical LV segment. Perivascular edema with single mononuclear cells is visible; (**H**) TTSO, 12 h post-ISO, mid-ventricular LV segment. Endocardial protrusions; (**I**) TTSO, 24 h post-ISO, apical LV segment. Fulminant course of Takotsubo syndrome. Severe interstitial and perivascular edema and diffuse infiltration of inflammatory cells are present with formation of inflammatory foci; (**J**) TTSO, 24 h post-ISO, apical LV segment. Interstitial edema, FCI, and severe diffuse inflammatory infiltration are visible; (**K**) TTSO, 24 h post-ISO, basal RV segment. Perivascular infiltrations of mononuclear cells; (**L**) TTSO, 24 h post-ISO, mid-ventricular RV segment. Endocardial protrusion; (**M**) TTSO, 72 h post-ISO, mid-ventricular LV segment. Large foci of inflammatory and fibroblast-like cell infiltration and diffuse inflammatory infiltration are visible; (**N**) TTSO, 72 h post-ISO, basal LV segment. Foci of inflammatory and fibroblast-like cell infiltration; (**O**) TTSO, 72 h post-ISO, basal LV segment. Severe perivascular infiltrations of mononuclear cells as in arteritis; (**P**) TTSO, 72 h post-ISO, mid-ventricular LV segment. Endocardial protrusion and infiltration with inflammatory cells are visible; (**Q**) CO, 24 h post-0.9% NaCl, mid-ventricular LV segment. Normal myocardium is visible; (**R**) CO, 24 h post-0.9% NaCl, mid-ventricular LV segment. Normal myocardium is visible; (**S**) CO, 24 h post-0.9% NaCl, mid-ventricular LV segment. Normal artery; (**T**) CO, 24 h post-0.9% NaCl, mid-ventricular LV segment. Normal myocardium and endocardium are visible. CO—control ovariectomized Sprague Dawley female rats injected with 0.9% NaCl; TTSO—ovariectomized Sprague Dawley female rats with isoprenaline-induced Takotsubo syndrome; ISO—isoprenaline; LV—left ventricle; RV—right ventricle.

**Figure 2:**
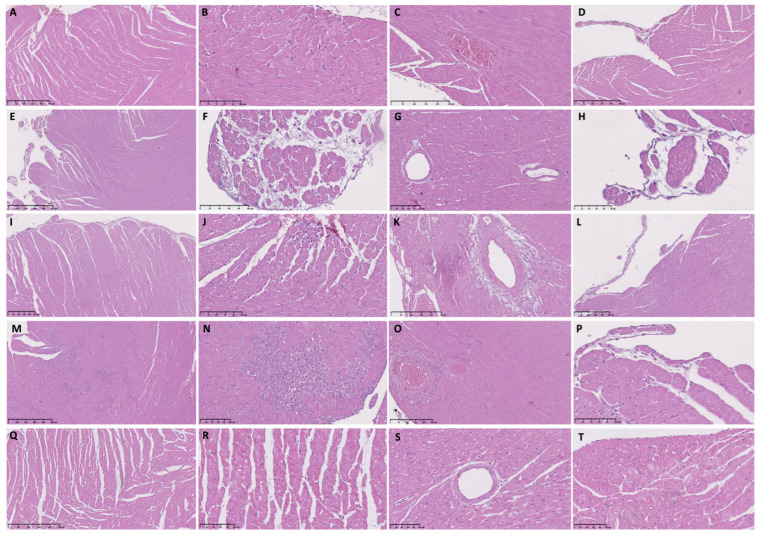
Histopathological findings in sham-operated female Sprague Dawley rats with Takotsubo syndrome and sham-operated control rats. (**A**) TTSP, 6 h post-ISO, mid-ventricular LV segment. Mild interstitial edema is visible; (**B**) TTSP, 6 h post-ISO, mid-ventricular LV segment. Interstitial edema and diffuse inflammatory infiltration are present; (**C**) TTSP, 6 h post-ISO, mid-ventricular RV segment. Normal artery is visible; (**D**) TTSP, 6 h post-ISO, basal RV segment. Endocardial protrusion; (**E**) TTSP, 12 h post-ISO, mid-ventricular LV segment. Interstitial, endocardial edema, and endocardial protrusions are present; (**F**) TTSP, 12 h post-ISO, mid-ventricular LV segment. Interstitial edema and sporadic single inflammatory cell infiltration are visible; (**G**) TTSP, 12 h post-ISO, apical LV segment. Slight perivascular edema with single mononuclear cells is visible; (**H**) TTSP, 12 h post-ISO, mid-ventricular LV segment. Endocardial protrusion; (**I**) TTSP, 24 h post-ISO, mid-ventricular LV segment. Interstitial and endocardial edema are visible; (**J**) TTSP, 24 h post-ISO, apical RV segment. FCI and interstitial edema are visible; (**K**) TTSP, 24 h post-ISO, basal LV segment. Perivascular edema and infiltrations similar to arteritis are visible; (**L**) TTSP, 24 h post-ISO, mid-ventricular RV segment. Endocardial protrusion; (**M**) TTSP, 72 h post-ISO, apical LV segment. Large foci of inflammatory and fibroblast-like cell infiltration and interstitial edema are visible; (**N**) TTSP, 72 h post-ISO, mid-ventricular LV segment. A large focal inflammatory and fibroblast-like cell infiltration is visible; (**O**) TTSP, 72 h post-ISO, mid-ventricular RV segment. Perivascular infiltrations of mononuclear cells resembling arteritis; (**P**) TTSP, 72 h post-ISO, mid-ventricular LV segment. Endocardial edema and protrusion are visible; (**Q**) CP, 24 h post-0.9% NaCl, mid-ventricular LV segment. Interstitial edema and inflammatory infiltration are visible; (**R**) CP, 24 h post-0.9% NaCl, mid-ventricular LV segment. Interstitial edema, vacuolization of cardiomyocytes, and inflammatory infiltration can be seen; (**S**) CP, 24 h post-0.9% NaCl, mid-ventricular LV segment. Slight perivascular edema with infiltration of mononuclear cells is present; (**T**) CP, 24 h post-0.9% NaCl, apical LV segment. Endocardium with features of slight edema, vacuolization, and mononuclear cells infiltrations are visible. CP—control sham-operated Sprague Dawley female rats injected with 0.9% NaCl; TTSP—sham-operated Sprague Dawley female rats with isoprenaline-induced Takotsubo syndrome; ISO—isoprenaline; LV—left ventricle; RV—right ventricle.

Six hours post-ISO injection, we found similar histopathological changes in the TTSO and TTSP rats. Endocardial and perivascular edema in the TTSP rats was visible only in the LV (Table 2, Figure 1A–D and Figure 2A–D).

Twelve hours post-ISO injection, several abnormalities, including cardiomyocyte vacuolization, blurred contours of cardiomyocytes, and perivascular edema, were visible only in the LV in the TTSO or TTSP rats. Endocardial protrusions were found in the LV and RV in the TTSO rats, whereas only in the LV in the TTSP rats (Table 2, Figure 1E–H and Figure 2E–H).

Twenty-four hours post-ISO injection, we observed substantial differences between TTSO and TTSP. The fulminant course of TTS in the TTSO rats, characterized by the presence of at least 10 FCI i.f.v., was visible. Rats from the TTSO group, in contrast with the TTSP rats, had severe LV diffuse inflammatory infiltration, interstitial and endocardial edema, and hemorrhages. In the LV in the TTSP rats, we also observed cardiomyocyte edema and vacuolization (Table 2, Figure 1I–L and Figure 2I–L).

In general, 72 h post-ISO injection, similar histopathological abnormalities were observed in the TTSO and TTSP rats. At FCI sites, the infiltration of fibroblast-like and inflammatory cells was visible. Only in the TTSO rats, the presence of endocardial edema and hemorrhages was observed (Table 2, Figure 1M–P and Figure 2M–P).

Moreover, after 6 and 12 h post-ISO injection, we observed perivascular edema and, 12 h post-ISO injection, with single mononuclear cells. Most interestingly, after 24 h post-ISO injection, we observed features of periarteritis, mainly concerning small and medium-sized arteries (Table 2, Figure 1 and Figure 2).

In the CO group, normal myocardium and vasculature of the heart was visible, with features of sporadic, rare, and small areas of cardiomyocyte vacuolization, slight interstitial and endocardial edema, blurred contours of the cardiomyocytes, and diffuse inflammatory infiltration (Figure 1Q–T)). On the other hand, intracellular, interstitial, endocardial, and perivascular edema were observed in the CP group, apart from vacuolization and blurred contours of the cardiomyocytes and diffuse infiltration of the inflammatory cells (Table 2, Figure 2Q–T).

### 3.3. Plasma Concentrations of TnI and NT-proBNP in the TTSO and TTSP Rats

Plasma concentrations of TnI were significantly higher in the TTSP group in comparison with the TTSO group (222.56 ± 15.03 vs. 120.01 ± 6.18 ng/L, *p* < 0.001). However, the rats from the TTSO group had a significantly higher plasma concentration of NT-proBNP in comparison with the TTSP group (1015.17 ± 27.75 vs. 173.42 ± 5.60 ng/L, *p* < 0.001) (Table 1).

### 3.4. Plasma Concentrations of Necroptosis and Pyroptosis-Associated Proteins in the TTSO and TTSP Rats

Plasma concentrations of necroptosis (CAMKIIδ, RIP3, MLKL) and pyroptosis (Casp-1, IL-1β, IL-18)-associated proteins in the TTSO, TTSP, and control groups are presented in Table 3. All TTS rats had a significantly higher plasma concentration of IL-1β when compared with the controls.

### 3.5. Myocardial Expression of Necroptosis and Pyroptosis-Associated Proteins in the TTSO and TTSP Rats

#### 3.5.1. Necroptosis-Associated Protein Expression

**Figure 3:**
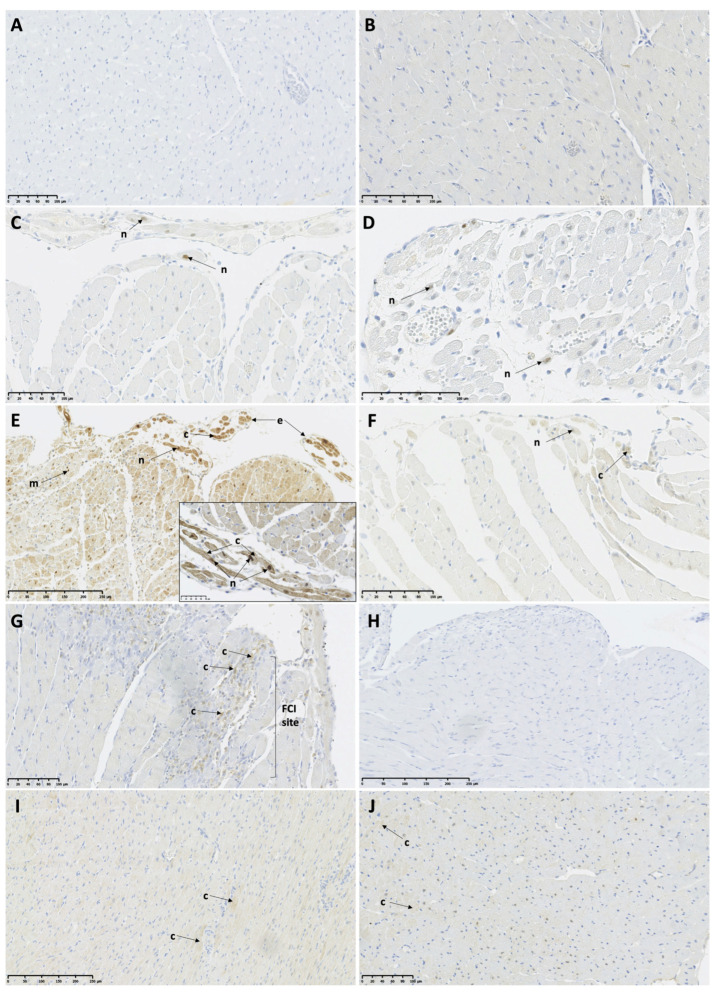
Calcium/calmodulin protein kinase II protein expression in female Sprague Dawley rats with Takotsubo syndrome and controls. (**A**) TTSO, 6 h post-ISO, mid-ventricular LV segment. No visible cardiomyocyte or mononuclear cell reaction; (**B**) TTSP, 6 h post-ISO, mid-ventricular LV segment. No visible cardiomyocyte or mononuclear cell reaction; (**C**) TTSO, 12 h post-ISO, mid-ventricular LV segment. Low protein expression localized in single cardiomyocyte nuclei; (**D**) TTSP, 12 h post-ISO, mid-ventricular LV segment. Low protein expression localized in single cardiomyocyte nuclei; (**E**) TTSO, 24 h post-ISO, mid-ventricular LV segment. High cytoplasmic and nuclear reaction in cardiomyocytes in remote myocardium and endocardial protrusions, high expression in mononuclear cells; (**F**) TTSP, 24 h post-ISO, mid-ventricular LV segment. Low protein expression localized in single cardiomyocyte nuclei and cytoplasm; (**G**) TTSO, 72 h post-ISO, mid-ventricular LV segment. Low protein expression localized in the FCI; (**H**) TTSP, 72 h post-ISO, mid-ventricular LV segment. No visible cardiomyocyte or mononuclear cell reaction; (**I**) CO, mid-ventricular LV segment. Low cardiomyocyte reaction; (**J**) CP, mid-ventricular LV segment. Low cardiomyocyte reaction is visible. CO—control ovariectomized Sprague Dawley female rats injected with 0.9% NaCl; CP—control sham-operated Sprague Dawley female rats injected with 0.9% NaCl; TTSO—ovariectomized Sprague Dawley female rats with isoprenaline-induced Takotsubo syndrome; TTSP—sham-operated Sprague Dawley female rats with isoprenaline-induced Takotsubo syndrome; c—cardiomyocyte cytoplasm; e—endocardial protrusion; FCI—foci of cardiomyocyte injury; ISO—isoprenaline; LV—left ventricle; m—mononuclear cell; n—cardiomyocyte nucleus; RM—remote myocardium; RV—right ventricle.

At 6 h post-ISO, CAMKIIδ protein expression was visible only in a few infiltrating mononuclear cells (Figure 3A,B; Table 4). At 12 h post-ISO, only low expression was observed in the FCI and the remote myocardium (RM) of both TTS groups (Figure 3C,D; Table 4). Most interestingly, at 24 h post-ISO, the TTSO rats showed high CAMKIIδ protein expression in the FCI and RM of both the LV and RV and in the vessels, while the TTSP rats showed only low protein expression equal to the 12 h post-ISO level. The intensity of expression showed a gradient, with the highest expression in the endocardium. The high expression of CAMKIIδ was visible also in the endocardial protrusions in the TTSO rats. On the other hand, the TTSP rats showed only a low protein expression of CAMKIIδ in limited locations of the myocardium and very small residual expression in the FCI (Figure 3E,F; Table 4). After 72 h post-ISO, a low residual CAMKIIδ protein expression was maintained within the sites of the FCI in the TTSO rats, while there was no visible immunohistochemical staining in the myocardium in the TTSP rats (Figure 3G,H; Table 4). On the contrary, the control groups, CO and CP, showed only a low sporadic protein expression of CAMKIIδ within the RM and vessels (V) (Table 4).

**Figure 4:**
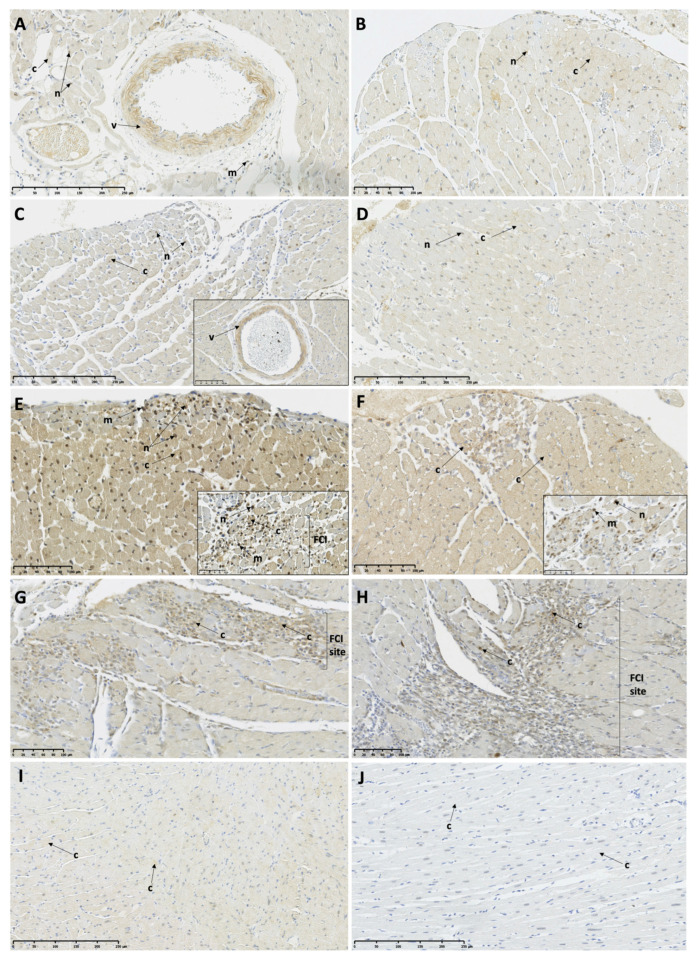
Mixed lineage kinase domain-like pseudokinase protein expression in female Sprague Dawley rats with Takotsubo syndrome and controls. (**A**) TTSO, 6 h post-ISO, mid-ventricular LV segment. High vascular and low cardiomyocyte cytoplasmic, nuclear, and mononuclear cell protein expression are visible; (**B**) TTSP, 6 h post-ISO, mid-ventricular LV segment. Low protein expression in cardiomyocyte cytoplasm and moderate in nuclei; (**C**) TTSO, 12 h post-ISO, mid-ventricular LV segment. Low protein expression localized in cardiomyocyte nuclei and cytoplasm, single infiltrating inflammatory cells, and vascular protein expression; (**D**) TTSP, 12 h post-ISO, mid-ventricular LV segment. Protein expression localized in cardiomyocyte nuclei and cytoplasm, as well as in single infiltrating inflammatory cells; (**E**) TTSO, 24 h post-ISO, mid-ventricular LV segment. High cytoplasmic and nuclear reaction in cardiomyocytes in FCI and RM, as well as high mononuclear cell reaction, are visible; (**F**) TTSP 24 h post-ISO, mid-ventricular LV segment. Low to moderate protein reaction in cytoplasm and high in nuclei of cardiomyocytes and mononuclear cells in FCI, low in cardiomyocyte cytoplasm in RM; (**G**) TTSO, 72 h post-ISO, mid-ventricular LV segment. Low protein expression in cytoplasm of cardiomyocytes in RM, high expression in mononuclear and fibroblast-like cells in FCI; (**H**) TTSP, 72 h post-ISO, mid-ventricular LV segment. Low cytoplasmic reaction of cardiomyocytes in RM, high cytoplasmic reaction in mononuclear and fibroblast-like cells in FCI; (**I**) CO, mid-ventricular LV segment. Low cardiomyocyte protein expression; (**J**) CP, mid-ventricular LV segment. No cardiomyocyte reaction is visible. CO—control ovariectomized Sprague Dawley female rats injected with 0.9% NaCl; CP—control sham-operated Sprague Dawley female rats injected with 0.9% NaCl; TTSO—ovariectomized Sprague Dawley female rats with isoprenaline-induced Takotsubo syndrome; TTSP—sham-operated Sprague Dawley female rats with isoprenaline-induced Takotsubo syndrome; c—cardiomyocyte cytoplasm; FCI—foci of cardiomyocyte injury; ISO—isoprenaline; LV—left ventricle; m—mononuclear cell; n—cardiomyocyte nucleus; RM—remote myocardium; RV—right ventricle.

After 6 and 12 h post-ISO, the MLKL protein showed low expression in some nuclei of the RV cardiomyocytes and infiltrating mononuclear cells in both the TTSO and TTSP rats. Vascular expression was high 6 h post-ISO in the TTSO rats and was low in the TTSP rats at 6 and 12 h post-ISO (Figure 4A–D; Table 4). On the contrary, after 24 h post-ISO, MLKL expression was high in the FCI, the myocardium of the RV and LV, and V and the infiltrating mononuclear cells in both the TTSO and TTSP rats (Figure 4E,F; Table 4). After 72 h post-ISO, high expression of MLKL was observed in some sites of the FCI, with low expression in some V, RM, and some infiltrating mononuclear cells (Figure 4G,H; Table 4). The CO group showed only low sporadic MLKL myocardial protein expression, while there was no protein expression of MLKL in the CP rats (Table 4).

No protein expression of RIP3 was observed in the TTSO, TTSP, and control rats, CO and CP (Table 4).

#### 3.5.2. Pyroptosis-Associated Protein Expression

**Figure 5:**
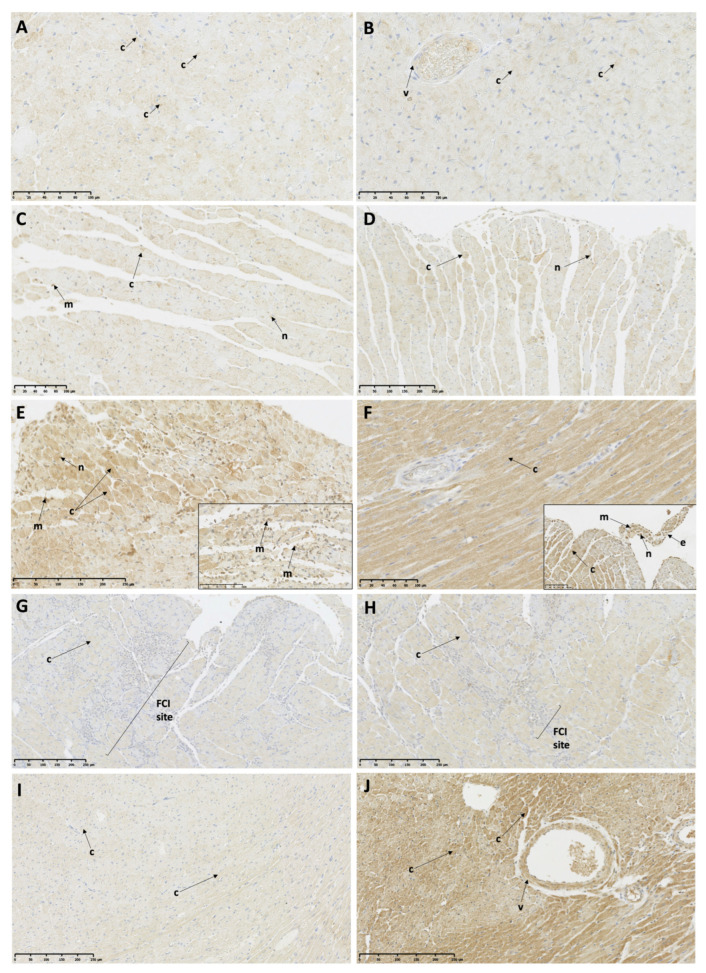
Caspase-1 protein expression in female Sprague Dawley rats with Takotsubo syndrome and controls. (**A**) TTSO, 6 h post-ISO, mid-ventricular LV segment. Low protein expression in cardiomyocyte cytoplasm is visible; (**B**) TTSP, 6 h post-ISO, mid-ventricular LV segment. Low protein expression in cardiomyocyte cytoplasm is visible; (**C**) TTSO, 12 h post-ISO, mid-ventricular LV segment. Low protein expression localized in cardiomyocyte cytoplasm, nuclei, and mononuclear cells; (**D**) TTSP, 12 h post-ISO, mid-ventricular LV segment. Low protein expression localized in cardiomyocyte nuclei and cytoplasm; (**E**) TTSO, 24 h post-ISO, mid-ventricular LV segment. High reaction in cytoplasm and nuclei of cardiomyocytes and mononuclear cells is visible; (**F**) TTSP, 24 h post-ISO, mid-ventricular LV segment. High reaction in cytoplasm and nuclei of cardiomyocytes and mononuclear cells, including endocardial protrusion; (**G**) TTSO, 72 h post-ISO, mid-ventricular LV segment. Low cytoplasmic reaction in RM and high reaction in cells in FCI; (**H**) TTSP, 72 h post-ISO, mid-ventricular LV segment. Moderate/high cytoplasmic reaction in RM and FCI; (**I**) CO, mid-ventricular LV segment. Low cardiomyocyte protein expression; (**J**) CP, mid-ventricular LV segment. High cardiomyocyte and vascular reaction is visible. CO—control ovariectomized Sprague Dawley female rats injected with 0.9% NaCl; CP—control sham-operated Sprague Dawley female rats injected with 0.9% NaCl; TTSO—ovariectomized Sprague Dawley female rats with isoprenaline-induced Takotsubo syndrome; TTSP—sham-operated Sprague Dawley female rats with isoprenaline-induced Takotsubo syndrome; c—cardiomyocyte cytoplasm; FCI—foci of cardiomyocyte injury; ISO—isoprenaline; LV—left ventricle; m—mononuclear cell; n—cardiomyocyte nucleus; RM—remote myocardium; RV—right ventricle.

At 6 and 12 h post-ISO, low protein expression of Casp-1 was observed in some cardiomyocytes and in the infiltrating mononuclear cells, as well as in the endocardial protrusions in the TTSO and TTSP rats (Figure 5A–D; Table 4). After 24 h post-ISO, high cytoplasmic expression of Casp-1 in the majority of the cardiomyocytes was observed in both the TTSO and TTSP rats, as well as in the FCI, the endocardial protrusions, and the infiltrating mononuclear cells (Figure 5E,F; Table 4). At 72 h post-ISO, low expression of Casp-1 was visible within some of the cardiomyocytes, V, and sites of the FCI (Figure 5G,H; Table 4). The CO group showed low Casp-1 myocardial protein expression, while CP rats showed high protein expression in myocardium and vessels (Table 4).

## 4. Discussion

The current study is a continuation of our previous reports regarding a female rat model of TTS, where we observed apoptosis in cardiomyocytes 24 h after ISO administration and presence of mild fibrosis, a characteristic feature of TTS, during both the acute and recovery phases [13,26]. To the best of our knowledge, this is the first study showing the activation of necroptosis and pyroptosis pathways in the rat model of ISO-induced TTS. We have shown the dynamic expressions of proteins involved in necroptosis and pyroptosis pathways, with concomitant release of inflammatory-related cytokines to the bloodstream. As mentioned above, the course of TTS is characterized by myocardial infiltration with inflammatory cells and extensive edema, increased levels of reactive oxygen species, nitrosative stress, and fibrosis, with an increase in systemic proinflammatory cytokines [18,27]. The increased level of proinflammatory cytokines persists for several months after the onset of TTS symptoms, which suggests a low-grade chronic inflammatory state [18]. Moreover, residual high systemic inflammation, defined as increased levels of C-reactive protein at discharge, is a predictor of incomplete recovery after the acute phase of TTS and is an independent factor of cardiovascular events [20,28]. Recently, it has been shown that patients with TTS have higher ultra-small iron oxide superparamagnetic particle (USPIO) retention in the LV during the acute phase, regardless of the ballooning or non-ballooning segment, when compared with the control group. USPIOs are only phagocytosed by activated tissue macrophages, therefore it has been assumed that macrophages are the main promoters of myocardial inflammation in the acute phase of TTS, which has also been confirmed in the post-mortem histopathological analysis of human hearts of patients who died during the acute phase of TTS [18,29]. Several mechanisms responsible for the increased myocardial inflammation have been proposed, such as direct myocyte injury or myocardial ischemia caused by excessive adrenergic stimulation, ROS production, and factors released by the neurons during intense adrenergic activation in the heart [27]. The current study sheds new light on the inflammatory process in TTS and suggests that programmed cell death may also serve as a primary trigger for inflammation in TTS.

The pathological increase in the concentration of circulating catecholamines and their excessive release from the cardiac sympathetic nerve endings lead to direct damage to the cardiomyocytes [30]. The pathophysiological mechanisms involved in catecholamine-induced myocardial injury include increased oxygen demand of the cardiomyocytes with insufficient supply, oxidative stress, calcium overload, myofibril over-contraction, and upregulation of the expression and release of inflammatory cytokines [31,32,33,34,35,36,37,38,39,40,41,42]. Indeed, histopathological examinations of the myocardium of rats treated with supraphysiological doses of catecholamines showed focal necrosis and degeneration of the cardiomyocytes, disordered myofibrils, advanced cytoplasmic vacuolization, myocardial infiltration with inflammatory cells, and tissue fibrosis [43,44,45]. Necrosis is the major pathological feature of catecholamine-induced myocardial injury [42,46]. However, recently, Wu et al. (2021) have proven that, in adult mixed-sex mice injected subcutaneously with ISO, a large proportion of cardiomyocyte necrosis induced by ISO is driven by necroptosis and is mediated by the RIPK1–RIPK3–MLKL pathway. Moreover, these authors have proven that targeting RIPK1 or RIPK3 can significantly attenuate ISO-induced necrosis [42]. To the best of our knowledge, the activation of the necroptosis pathway has not yet been investigated in TTS. Therefore, this is the first study that shows high expression of necroptosis-associated proteins, including MLKL and CAMKII in ISO-induced TTS in female rats. Based on the available data, it can be concluded that CAMKII is a protein directly involved in cardiomyocyte apoptosis caused by stimulation of β1-adrenergic receptors and involved in cardiomyocyte necroptosis with accompanying inflammation. Moreover, the expression of CAMKII increases with low levels of estrogens [47,48,49,50]. CAMKII is a serine/threonine kinase activated by the increase in the concentration of calcium ions within the cell [51]. The CAMKII delta isoform is found mainly in the heart and vessels (CAMKIIδ) [51]. Zhang et al. (2016) showed that RIP3 triggers myocardial necroptosis in addition to apoptosis and inflammation through the activation of CAMKII, rather than through the RIP1 and MLKL proteins. These authors showed that, in mice treated with doxorubicin or subjected to ischemia/reperfusion, RIP3 deficiency or CAMKII inhibition alleviates myocardial necroptosis and HF [49]. In our study, 24 h post-ISO, a high expression of CAMKII was visible in the myocardium and vessels of TTSO rats, while in the TTSP rats only low protein expression was visible. In contrast, the expression of MLKL was equally high in both the TTSO and TTSP groups. Therefore, we hypothesized that, apart from MLKL-dependent necroptosis, reduced levels of female sex hormones may enhance the expression of CAMKII in the cardiomyocytes and therefore lead to CAMKII-associated necroptosis in response to a catecholamine surge, which may contribute to the fulminant course of the disease. Nevertheless, the exact mechanism of cardiomyocyte necroptosis in TTS and its impact on the course of the disease needs to be determined in further experimental studies.

In 2017, Xiao et al. proved that ISO administration in male C57BL/6 mice activates inflammasome and IL-18 in cardiomyocytes, which therefore initiates an inflammatory response within the heart [52]. Further study confirmed that Casp-1 cleavage increases in isolated neonatal mouse cardiomyocytes, even 15 minutes after administration of ISO, and is sustained for up to 1 hour post-ISO, which suggests that ISO may directly mobilize inflammasomes in the cardiomyocytes. Moreover, the authors proved that ISO induces pyroptosis in isolated mouse neonatal cardiomyocytes and cardiac fibroblasts connected to cardiomyocytes by membrane nanotubules, even after 1 hour post-ISO administration [53]. In this study, we showed high cytoplasmic expression of Casp-1 in the majority of cardiomyocytes, irrespective of whether the rats had an ovariectomy. As mentioned earlier, pyroptosis results in instantaneous cell membrane disruption and the release of cellular contents with inflammatory cytokines, including IL-1β and IL-18, to the bloodstream [5]. Indeed, we have proven that plasma concentrations of IL-1β are higher in the TTS groups when compared with the controls. Mature IL-1β serves as a proinflammatory cytokine that recruits innate immune cells to the location of inflammation and modulates adaptive immune cells, including T helper 1 (Th) and Th 17 [5]. Therefore, the results of this study suggest that the programmed death of cardiomyocytes associated with inflammation, i.e., necroptosis and pyroptosis, may be responsible in part for the inflammatory response in the course of TTS.

As first, we compared the course of specifically ISO-induced TTS between ovariectomized and fertile female rats and proved that ovariectomized rats have extremely advanced myocardial degeneration and that, after 24 h post-ISO, the course of TTS is fulminant with a highly advanced inflammatory response. Moreover, even after 6 h post-ISO, we showed that the FCI, diffuse inflammatory infiltration, interstitial, cardiomyocyte, perivascular and endocardial edema, as well as cardiomyocyte vacuolization, are present. Ovariectomized rats also have significantly higher LVEDD and LVEDA values with higher plasma concentrations of NT-proBNP, which indicates a more advanced LV volume overload and dilatation. In fertile female Sprague Dawley rats with TTS induced by 150 mg/kg b.wt ISO, after 24 h post-ISO, Kołodzińska et al. (2020) showed the presence of focal cardiomyocyte necrosis/apoptosis with inflammatory and fibroblast-like cell infiltration, vacuolization of cardiomyocytes, and interstitial edema; while, after 48 h post-ISO, the following were observed: larger foci of inflammatory and fibroblast-like cell infiltration, without cardiomyocyte necrosis or apoptosis, and cardiomyocyte vacuolization, with less interstitial edema and small focal hemorrhages [25]. In a study on Sprague Dawley rats, Shao et al. (2013) injected i.p. 50–600 mg/kg b.wt ISO and observed typical LV apical ballooning, even after 2 h post-ISO administration, and revealed severe lipid accumulation in the akinetic apical segments with small lipid accumulation in the hyperkinetic basal segments of LV [50]. Iacucci et al. (2013), in a study on the myocardial biopsies from patients with an acute phase of TTS, reported the presence of contraction band necrosis, massive interstitial edema, and infiltration of inflammatory cells [54]. Moreover, Elsokkari et al. (2013), in a post-mortem analysis of a female patient with TTS, reported the presence of scattered local myocardial necrosis with surrounding lymphocytes, hemorrhages, and infiltration with inflammatory cells [55]. Both in the TTSO and TTSP rats, we also observed the presence of arteritis in small and medium-sized arteries, which was previously reported in patients with TTS [56]. Interestingly, the endothelium of these arteries showed a high expression of necroptosis and pyroptosis-associated proteins. Akashi (2005) proposed that polyangiitis may lead to the failure of the coronary microcirculation, which therefore could be responsible for the reversible LV dysfunction [57]. Therefore, our study appears to be in line with this hypothesis, suggesting that the probable occurrence of necroptosis and pyroptosis is the mechanism for endothelial cell damage and the accompanying vascular inflammatory reaction.

During the course of ISO-induced TTS, we observed the development of endocardial protrusions with a high expression of necroptosis and pyroptosis-associated proteins. Due to their narrow stalk and potential mobile character, these protrusions can detach in vivo and, with a high probability, can serve as a source of embolic material. Indeed, clinical studies have shown higher stroke rates in TTS when compared with acute myocardial infarction [58]. A recent literature review revealed that, of 282 patients with TTS, 26 were found to have a thromboembolic event; however, 10 of them had no evidence of LV thrombus [59]. There are also several cases of patients with TTS, complicated by embolism (renal, cerebral), in which the presence of LV thrombus was excluded [60,61]. Therefore, we hypothesize that necroptosis and pyroptosis may cause endocardial fragmentation, which could be associated with the peripheral embolism during the course of TTS.

One of the characteristic features of TTS is that there is only a moderate increase in plasma TnI concentration, much lower than in acute myocardial infarction [62]. We have also observed relatively lower plasma concentrations of TnI when compared with other studies of ISO-induced myocardial injury. Our histopathological analysis, in agreement with other studies of experimental animal models and clinical cases of TTS, showed only the presence of focal injury of cardiomyocytes scattered between normal cardiomyocytes, therefore the area of myocardial necrosis was relatively small [21,26,63]. A hypothetical mechanism that may be responsible for the lower plasma concentration of TnI in TTS is the dominance of the programmed type of cardiomyocyte cell death. So far, myocardial necrosis has been considered to be the most obvious cause of the increase in plasma Tn levels, especially when the increase in plasma Tn levels is transient [64]. Recent reports indicate, however, that in the ischemic myocardium, the pathways of programmed cardiomyocyte death, including necroptosis, are activated [64]. In the course of necroptosis, there is a loss of the continuity of the cytoplasmic membrane, edema, and cell lysis, which presumably results in the release of Tn into the extracellular space, but this has not yet been fully confirmed [3,64]. It is emphasized in the literature that the dynamics of Tn release may be different in the case of necroptosis when compared with the necrosis of cardiomyocytes [64]. Necroptosis may be delayed after an ischemic event and leads to a gradual release and prolonged elevation of plasma Tn [64]. Therefore, we hypothesize that the presence of necroptosis or pyroptosis in TTS results in a relatively low but gradual and sustained release of TnI into the plasma, which may explain different plasma concentrations of troponins in patients with TTS when compared with acute myocardial infarction; however, this suggestion needs to be confirmed in further experimental studies.

## 5. Conclusions

So far, it has not been clear whether inflammation is the pathophysiological mechanism responsible for the pathogenesis of TTS or whether it is only a result of tissue injury. We have demonstrated the activation of inflammatory-associated programmed cell death, necroptosis, and pyroptosis in TTS, therefore it may be assumed that inflammation is a primary response occurring simultaneously with cardiomyocyte cell death in TTS. Programmed cell death may explain the relatively low concentrations of troponins during the acute phase of TTS. Moreover, we suggest that necroptosis and pyroptosis may cause the potential fragmentation of the endocardium, which may be the cause of peripheral embolism of an unclear source observed in patients. These results also suggest the probable occurrence of necroptosis and pyroptosis as a mechanism for endothelial cell damage and the accompanying vascular inflammatory reaction, which also supports the hypothesis that arteritis of small and medium-sized vessels is associated with TTS.

## 6. Study Limitations

A limitation of our study was the lack of a control group of ovariectomized female rats receiving supplementation of 17-beta estradiol. In this study, the histopathological analysis showed signs of myocardial damage also in animals from the CO and CP groups. Abnormalities in the structure of the myocardium was observed using light microscopy with increased plasma concentrations of TnI in the control Sprague Dawley rats, which were not subjected to any experimental procedure, as shown previously [65]. It would therefore appear that emotional stress and stress resulting from the ovariectomy and sham surgery could induce TTS in some of the control animals. Moreover, reliable statistical analysis of comparison between different time points after ISO administration cannot be conducted due to an insufficient number of individuals in particular subgroups (less than three).

## Figures and Tables

**Table 1:** Echocardiographic parameters of cardiac function and plasma level of cardiac biomarkers in ovariectomized and fertile female Sprague Dawley rats with isoprenaline-induced Takotsubo syndrome and controls.

Analyzed Parameter	TTSO	CO	*p*-Value *	TTSP	CP	*p*-Value **	*p*-Value ***
Left ventricular dimensions and systolic function							
LVEDD (cm)	0.65 ± 0.02	0.51 ± 0.04	<0.01	0.58 ± 0.01	0.45 ± 0.03	**<0.01**	**<0.01**
LVEDA (cm^2^)	0.63 ± 0.01	0.52 ± 0.04	<0.05	0.54 ± 0.02	0.52 ± 0.03	NS	**<0.01**
LVESA (cm^2^)	0.17 ± 0.01	0.19 ± 0.02	NS	0.2 ± 0.02	0.16 ± 0.01	NS	NS
FAC (%)	72.23 ± 2.33	64.3 ± 1.54	<0.05	63.94 ± 2.82	68.85 ± 2.33	NS	**<0.05**
FS (%)	54.49 ± 3.07	48.59 ± 4.95	NS	51.2 ± 1.42	49.45 ± 2.18	NS	NS
Plasma concentration of cardiac biomarkers							
TnI (ng/L)	120.01 ± 6.18	135.6 ± 10.59	NS	222.56 ± 15.03	178.26 ± 4.73	NS	**<0.001**
NT-proBNP (ng/L)	1015.17 ± 27.75	894.19 ± 31.05	NS	173.42 ± 5.60	478.77 ± 30.53	**<0.001**	**<0.001**

CO—control ovariectomized Sprague Dawley female rats injected with 0.9% NaCl; CP—control sham-operated Sprague Dawley female rats injected with 0.9% NaCl; TTSO—ovariectomized Sprague Dawley female rats with isoprenaline-induced Takotsubo syndrome; TTSP—sham-operated Sprague Dawley female rats with isoprenaline-induced Takotsubo syndrome; FAC—fractional area change; FS—fractional shortening; LVEDA—left ventricular end-diastolic area; LVEDD—left ventricular end-diastolic dimension; LVESA—left ventricular end-systolic area; NS—nonsignificant; NT-proBNP—N-terminal pro-brain natriuretic peptide; TnI—Troponin I. Continuous variables are presented as mean ± standard deviation. * *p*-value TTSO vs. CO; ** *p*-value TTSP vs. CP; *** *p*-value TTSO vs. TTSP.

**Table 2:** Histopathological analysis in female Sprague Dawley rats with isoprenaline-induced Takotsubo syndrome and controls.

	6 h Post-ISO				12 h Post-ISO				24 h Post-ISO				72 h Post-ISO				24 h Post-0.9% NaCl			
	TTSO		TTSP		TTSO		TTSP		TTSO		TTSP		TTSO		TTSP		CO		CP	
	LV	RV	LV	RV	LV	RV	LV	RV	LV	RV	LV	RV	LV	RV	LV	RV	LV	RV	LV	RV
Number of FCI	0–10	numerous/ some/only a few (3–5)	10–50	1–8	2–35	3–5	0–6	0–4	8–47	9–27	3–16	1–13	3–52 #	2–11 #	7–40 #	1–3 #	–	–	–	–
Mean number of FCI	6 ± 5	–	26 ± 19	5 ± 2	15 ± 18	–	–	–	22 ± 13	14 ± 6	8 ± 4	6 ± 4	24 ± 15	6 ± 3	20 ± 17	2 ± 1	–	–	–	–
Cardiomyocyte vacuolization	+	+	+	+	+	–	+	–	–	–	+	–	–	–	–	–	-/+	-/+	-/+	-/+
Diffuse inflammatory infiltration	+	+	+	+	+	+	+	+	++	+	+	+	+	+	+	+	-/+	-/+	-/+	-/+
Interstitial edema	+	+	+	+	+	+	+	+	++	++	+	+	+	+	+	+	-/+	-/+	-/+	-/+
Cardiomyocyte edema	–	–	–	–	–	–	–	–	–	–	+	+	+	+	+	+	–	–	-/+	-/+
Endocardial edema	+	+	+	–	+	+	+	+	++	++	+	+	+	+	–	–	-/+	-/+	-/+	-/+
Endocardial protrusions	+	+	+	+	+	+	+	–	+	+	+	+	+	+	+	+	–	–	–	–
Perivascular edema	+	+	+	–	+	–	–	–	+	–	+	–	+	–	+	+	–	–	-/+	-/+

CO—control ovariectomized Sprague Dawley female rats injected with 0.9% NaCl; CP—control sham-operated Sprague Dawley female rats injected with 0.9% NaCl; TTSO—ovariectomized Sprague Dawley female rats with isoprenaline-induced Takotsubo syndrome; TTSP—sham-operated Sprague Dawley female rats with isoprenaline-induced Takotsubo syndrome; ISO—isoprenaline; FCI—foci of cardiomyocyte injury; LV—left ventricle; RV—right ventricle; #—infiltration of fibroblast-like and inflammatory cells at FCI sites; “–“—absent; “+”—present with low/moderate severity; “++”—present with high severity (example Figure 1I,J); “-/+”—present with low/moderate severity only in a subset of cases.

**Table 3:** Plasma level of necroptosis and pyroptosis-related proteins in ovariectomized and fertile female Sprague Dawley rats with isoprenaline-induced Takotsubo syndrome.

Analyzed Protein	TTSO	TTSP	CO	CP	*p*-Value
Necroptosis-related proteins					
CAMKIIδ (ng/mL)	4 (3.88–5)	19 (18.16–19) *	2 (1.88–2) ###	19 (18.87–19)	<0.001
RIP3 (ng/mL)	0.75 (0.59–0.88)	0.99 (0.93–1.02)	0.95 (0.9–1.3)	1 (0.89–1.08)	NS
MLKL (ng/L)	359 (323–393)	744 (712–835) ***	357 (332–385)	585 (560–599)	<0.001
Pyroptosis-related proteins					
Casp-1 (ng/mL)	5.12 (4.93–5.38)	2.59 (2.40–2.80) ***	5.10 (5.00–5.21) #	2.54 (2.40–2.74)	<0.001
IL-1β (pg/L)	2016 ± 108	2778 ± 215 ###,**	1133 ± 197 **	1000 ± 63	<0.001
IL-18 (pg/mL)	39 (36–46)	44 (41–45)	45 (44–47)	47 (43–49)	NS

CO—control ovariectomized Sprague Dawley female rats injected with 0.9% NaCl; CP—control sham-operated Sprague Dawley female rats injected with 0.9% NaCl; TTSO—ovariectomized Sprague Dawley female rats with isoprenaline-induced Takotsubo syndrome; TTSP—sham-operated Sprague Dawley female rats with isoprenaline-induced Takotsubo syndrome; CAMKIIδ—calcium/calmodulin-dependent kinase type II isoform delta; Casp-1—caspase-1; IL-1β—interleukin-1β; IL-18—interleukin-18; MLKL—mixed lineage kinase domain-like pseudokinase; NS—nonsignificant; RIP3—receptor-interacting serine/threonine-protein kinase 3. Continuous variables are presented as median and interquartile range. * *p* < 0.05 vs. TTSO, ** *p* < 0.01 vs. TTSO, *** *p* < 0.001 vs. TTSO; # *p* < 0.05 vs. CP, ### *p* < 0.001 vs. CP.

**Table 4:** Necroptosis and pyroptosis-associated protein expression signal intensity during the acute phase of TTS in ovariectomized and fertile female Sprague Dawley rats.

	CAMKIIδ				MLKL					RIP3					Casp-1			
	TTSO	TTSP	CO	CP	TTSO		TTSP	CO	CP	TTSO		TTSP	CO	CP	TTSO	TTSP	CO	CP
6 h post-ISO	FCI-no RM-no V-no		FCI-no RM-no V-no	FCI-absent RM-low V-low	FCI-absent RM-low V-low	FCI-no RM-low V-high	FCI-no RM-low V-low		FCI-absent RM-low V-no	FCI-absent RM-no V-no	FCI-no RM-no V-no	FCI-no RM-no V-no	FCI-absent RM-no V-no	FCI-absent RM-no V-no	FCI-low RM-low V-low	FCI-low RM-low V-no	FCI-absent RM-low V-no	FCI-absent RM-high V-high
12 h post-ISO	FCI-low RM-low V-no		FCI-low RM-low V-no			FCI-no RM-low V-high	FCI-no RM-low V-low				FCI-no RM-no V-no	FCI-no RM-no V-no			FCI-low RM-low V-low	FCI-low RM-low V-low		
24 h post-ISO	FCI-high RM-high V-high		FCI-low RM-low V-no			FCI-high RM-high V-high	FCI-high RM-high V-high				FCI-no RM-no V-no	FCI-no RM-no V-no			FCI-high RM-high V-high	FCI-high RM-high V-low		
72 h post-ISO	FCI-low RM-low V-no		FCI-no RM-no V-no			FCI-high RM-low V-low	FCI-high RM-low V-low				FCI-no RM-no V-no	FCI-no RM-no V-no			FCI-low RM-low V-low	FCI-low RM-low V-low		

TTSO—ovariectomized Sprague Dawley female rats with isoprenaline-induced Takotsubo syndrome; TTSP—sham-operated Sprague Dawley female rats with isoprenaline-induced Takotsubo syndrome; CO—control ovariectomized Sprague Dawley female rats injected with 0.9% NaCl; CP—control sham-operated Sprague Dawley female rats injected with 0.9% NaCl; ISO—isoprenaline; FCI—foci of cardiomyocyte injury; RM—remaining myocardium; V—vessels; CAMKIIδ—calcium/calmodulin-dependent kinase type II isoform delta; Casp-1—caspase-1; MLKL—mixed lineage kinase domain-like pseudokinase; RIP3—receptor-interacting serine/threonine-protein kinase 3. “no”—no visible protein expression (examples Figure 3A,B,H); “low”—low protein expression signal intensity (examples Figure 3C,D,F,G); “high”—high protein expression signal intensity (examples Figure 3E and Figure 4E).

## Data Availability

All data analyzed during this study are included in this published article and its supplementary information files.

## Supplementary Materials

The following supporting information can be downloaded at: https://www.mdpi.com/article/10.3390/biomedicines11072060/s1.
