# Supplementary material for: Inflammatory Forms of Cardiomyocyte Cell Death in the Rat Model of Isoprenaline-Induced Takotsubo Syndrome
Source: Biomedicines. 2023 Jul 21;11(7):2060. doi: 10.3390/biomedicines11072060 (PMC10377582; doi:10.3390/biomedicines11072060)
Supplement: Supplementary file 1 [file biomedicines-11-02060-s001.zip › biomedicines-2485124-supplementary.pdf]

Supplementary Table S1. Echocardiographic parameters of cardiac function in ovariectomized and fertile female Sprague Dawley rats with isoprenaline-induced Takotsubo syndrome in different time points after isoprenaline administration.

Parameter	Time post-ISO				p-value
	6h	12h	24h	72h	
LVEDD (cm)	0.58±0.03	0.62±0.04	0.62±0.02	0.66±0.02	*NS*
LVEDA (cm^2)	0.55±0.03	0.60±0.11	0.59±0.02	0.62±0.02	*NS*
LVESA (cm^2)	0.15±0.02	0.19±0.03	0.21±0.02	0.19±0.03	*NS*
FAC (%)	73.40±2.63	66.74±10.95	64.03±2.86	68.86±5.30	*NS*

ISO – isoprenaline; FAC – fractional area change; LVEDA – left ventricular end-diastolic area; LVEDD – left ventricular end-diastolic dimension; LVESA – left ventricular end-systolic area; NS – non-significant. Continuous variables are presented as mean± standard deviation.
